# Molecular surveillance reveals the presence of *pfhrp2* and *pfhrp3* gene deletions in *Plasmodium falciparum* parasite populations in Uganda, 2017–2019

**DOI:** 10.1186/s12936-020-03362-x

**Published:** 2020-08-26

**Authors:** Agaba B. Bosco, Karen Anderson, Karryn Gresty, Christiane Prosser, David Smith, Joaniter I. Nankabirwa, Sam Nsobya, Adoke Yeka, Jimmy Opigo, Samuel Gonahasa, Rhoda Namubiru, Emmanuel Arinaitwe, Paul Mbaka, John Kissa, Sungho Won, Bora Lee, Chae Seung Lim, Charles Karamagi, Jane Cunningham, Joan K. Nakayaga, Moses R. Kamya, Qin Cheng

**Affiliations:** 1 College of Health Sciences, Makerere University, Kampala, Uganda; 2 National Malaria Control Division, Kampala, Uganda; 3 QIMR Berghofer Medical Research Institute, Brisbane, QLD Australia; 4 Australian Defence Force Malaria and Infectious Disease Institute, Enoggera, Australia; 5 Infectious Diseases Research Collaboration, Kampala, Uganda; 6 World Health Organization Country Office, Kampala, Uganda; 7 National Health Information Division, Ministry of Health, Kampala, Uganda; 8 Department of Public Health Sciences, Seoul National University, Seoul, South Korea; 9 Department of Laboratory Medicine, College of Health Sciences, Korea University, Seoul, South Korea; 10 World Health Organization, Geneva, Switzerland

**Keywords:** Malaria rapid diagnostic tests, *Plasmodium falciparum*, Histidine rich protein 2, Histidine rich protein 3, Gene deletion, Deoxyribonucleic acid, Microscopy

## Abstract

**Background:**

Histidine-rich protein-2 (HRP2)-based rapid diagnostic tests (RDTs) are the only RDTs recommended for malaria diagnosis in Uganda. However, the emergence of *Plasmodium falciparum* histidine rich protein 2 and 3 (*pfhrp2* and *pfhrp3)* gene deletions threatens their usefulness as malaria diagnostic and surveillance tools. The *pfhrp2* and *pfhrp3* gene deletions surveillance was conducted in *P. falciparum* parasite populations in Uganda.

**Methods:**

Three-hundred (n = 300) *P. falciparum* isolates collected from cross-sectional malaria surveys in symptomatic individuals in 48 districts of eastern and western Uganda were analysed for the presence of *pfhrp2* and *pfhrp3* genes. Presence of parasite DNA was confirmed by PCR amplification of the *18s rRNA* gene*, msp1* and *msp2* single copy genes. Presence or absence of deletions was confirmed by amplification of exon1 and exon2 of *pfhrp2* and *pfhrp3* using gene specific PCR.

**Results:**

Overall, *pfhrp2* and *pfhrp3* gene deletions were detected in 29/300 (9.7%, 95% CI 6.6–13.6%) parasite isolates. The *pfhrp2* gene was deleted in 10/300 (3.3%, 95% CI 1.6–6.0%) isolates, *pfhrp3* in 9/300 (3.0%, 95% CI 1.4–5.6%) while both *pfhrp2* and *pfhrp3* were deleted in 10/300 (3.3%, 95% CI 1.6–6.0%) parasite isolates. Proportion of *pfhrp2/3* deletions was higher in the eastern 14.7% (95% CI 9.7–20.0%) compared to the western region 3.1% (95% CI 0.8–7.7%), p = 0.001. Geographical location was associated with gene deletions aOR 6.25 (2.02–23.55), p = 0.003.

**Conclusions:**

This is the first large-scale survey reporting the presence of *pfhrp2/3* gene deletions in *P. falciparum* isolates in Uganda. Roll out of RDTs for malaria diagnosis should take into consideration the existence of *pfhrp2/3* gene deletions particularly in areas where they were detected. Periodic *pfhrp2/3* surveys are recommended to inform future decisions for deployment of alternative RDTs.

## Background

In 2018, the World Health Organization (WHO) estimated there were 228 million cases and 405,000 deaths globally due to malaria. The WHO African Region continues to contribute a disproportionately high share of the global burden (93% of malaria cases and 94% of malaria deaths) in 2018 alone [1]. Nearly all malaria cases in the region are caused by *Plasmodium falciparum.* Uganda is ranked among the six highest burden countries [1]. The 2018 and 2014 Uganda national malaria indicator surveys have reported overall malaria parasite prevalence of 9 and 19%, respectively [3, 4]. *Plasmodium falciparum* is the most predominant parasite in Uganda, accounting for > 95% of malaria infections [3, 5].

The WHO recommends parasitological confirmation of malaria in all suspected cases prior to treatment with artemisinin-based combination therapy (ACT) [6, 7]. The Uganda National Malaria Control Division adopted this policy and shifted from clinical to parasite-based diagnosis with microscopy or rapid diagnostic tests (RDTs) in 2011 [6, 7]. Since the introduction of RDTs in late 2000s, over 800 million RDTs have been used for malaria testing in Uganda which has led to increased access to parasite-based diagnosis [2, 8]. A similar increase has been seen particularly in the African region, where large volumes of histidine rich protein 2 (HRP2)-based RDTs are used due to the predominance of *P. falciparum* species in this region [1, 2]. However, RDTs must remain effective and accurate in detecting the presence of parasites in order to be useful in supporting diagnostic and surveillance programmes [9–11]. There are several documented factors that have been known to affect the accuracy and functionality of RDTs that range from product design, transport or storage conditions, parasite factors due to gene deletions, operator-related factors and host parasite densities [12, 13]. Many endemic countries in collaboration with the WHO and the manufacturers have instituted quality assurance systems to address the possible causes of false RDT results [14, 15], however, parasite gene deletions have not been studied at a wider scale in many parts of Africa and evidence remains limited [12, 16]. Studies have suggested the possibility of evolution of gene-deleted parasites by a genetic event due to selective pressure resulting from long-term use of HRP2-based RDTs [17]. Failure of the parasite to express the HRP2 target antigen, alteration in the HRP2 protein sequence or pattern of histidine repeats and variation in the number of repeats have been known to affect the sensitivity of HRP2-based RDTs [18–20]. Although investigation of other causes of false negative RDTs was outside the scope of this study, several studies have provided possible explanations for their occurrences. They include variation in the composition of *pfhrp2* repeat sequence, number of repeat types and the amino acid composition of the HRP2 all of which may have impact on RDT sensitivity [19, 21].

Due to the predominance of *P. falciparum* in Uganda, the national policy recommends exclusive use of HRP2-based RDTs for malaria diagnosis [7]. The principal target recognized by HRP2-based RDTs are HRP2 antigens although, due to similarity in amino acid sequences, antibodies cross-react with HRP3 [5, 7]. These antigens are not expressed in malaria parasites in some parts of Africa due to the absence of the *pfhrp2* and *pfhrp3* genes [12, 16, 17, 19, 22–28]. When *P. falciparum* parasites express little or no *pfhrp2/3* target antigens, they are not detected by HRP2-based RDTs, threatening the usefulness of HRP2-based RDTs as a diagnostic test [11, 12, 25]. This poses a public health threat as a large number of infected patients will go untreated, leading to increased risk of malaria morbidity, mortality and transmission [12, 16, 17, 22].

The WHO recommends routine surveillance of *pfhrp2* and *pfhrp3* gene deletions in malaria parasites in countries that are neighbouring areas where deletions have been confirmed or where there are reports of false negative RDTs [9–11, 16]. Surveillance data on parasite gene deletions could potentially inform national malaria diagnostic policies regarding choice of RDTs [11, 12]. A policy switch to more effective, alternative RDTs is recommended when the prevalence of false negative RDT results due to *pfhrp2/3*- gene deletions exceeds 5% [10, 11]. A threshold of 5% was selected because it is somewhere around this point that the proportion of cases missed by HRP2 RDTs due to non-HRP2 expression may be greater than the proportion of cases that would be missed by less-sensitive pLDH-based RDTs [29].

Parasite *pfhrp2/3* gene deletions have been reported in areas neighbouring Uganda, including Kenya, Democratic Republic of Congo (DRC), Rwanda, and Eritrea [17, 28, 30, 31], however data on their occurrence and distribution in Uganda are limited. Only one study in Uganda reported the existence of *pfhrp2/3* gene deletions in nine (9/416) PCR-confirmed parasite isolates, however its scope was limited to archived samples in one district [32]. The magnitude, extent of spread and the possible factors associated with the *pfhrp2/3* gene deletions in Uganda is poorly understood. To improve understanding of the extent and spectrum of *pfhrp2* and *pfhrp3* gene deletions in Uganda on a wider scale, surveillance of *pfhrp2* and *pfhrp3* gene deletions was conducted in *P. falciparum* parasite populations in 48 districts of Uganda.

## Methods

### Study design and setting

This was a cross-sectional study that analysed samples collected from parasite surveys that were conducted in 48 out of 134 districts of Uganda between 2017 and 2019. The primary objective of the surveys was to evaluate the effect of piperonyl butoxide (PBO) long-lasting insecticide-treated nets (LLINs) on parasite prevalence [33, 34] and covered nearly half of the country and a wide range of epidemiological settings. Details of the PBO study have been published elsewhere [33, 34]. Malaria is endemic in 95% of the country, and transmission occurs throughout the year with two peak transmission seasons between June to July and November to December [5, 33, 34]. The parasite surveys were conducted at 6-month intervals and coincided with the two peak transmission seasons.

### Study population

Details of sampling, participant selection and enrolment have been described and published under the PBO studies [33, 34]. Briefly, a total of 104 clusters (Health Sub-Districts) across 48 districts were selected and randomized to receive different LLINs. Fifty (n = 50) households were randomly selected from each cluster. In selected households, children aged 2–10 years were assessed for presence of fever (based on axillary temperature of > 37.5 °C) before enrolment. Enrolled children were tested for malaria using RDTs and by malaria microscopy [33, 34]. Additionally, dried blood spots (DBS) were collected and stored for molecular testing of parasites. Written consent was obtained from all participants prior to study procedures commencing.

This study utilized the DBS samples from the PBO parasite surveys to assess the presence of *pfhrp2* and *pfhrp3* gene deletions. Included in the study were samples that were RDT negative but microscopy positive for malaria RDT−/microscopy+ (n = 222) and a random sample set of 15% (n = 140) from those that were both RDT and microscopy-positive (RDT+/microscopy+). The additional inclusion criteria were if the participants were aged 2–10 years, had a DBS filter paper sample available and provided consent for use of samples for future studies. Samples were excluded if they contained non-*P. falciparum* species by DNA PCR and if they had low quality DNA based on failure to amplify the single copy genes merozoite surface protein 1 (MSP1) and merozoite surface protein 2 (MSP2).

### Laboratory analysis

#### Rapid diagnostic tests (RDTs)

As part of the PBO parasite surveys, a HRP2-based *P. falciparum*-specific RDT (SD Bioline Malaria Ag Pf 05FK120; Standard Diagnostics, Gyeonhhi-do, South Korea) was used to test for malaria in febrile patients with a history of fever (based on axillary temperature of > 37.5 °C). The test is designed to exclusively detect *P. falciparum* infections only. RDT testing was done as per the manufacturer’s instructions. The RDT results were obtained from the PBO study database.

#### Blood smear microscopy

In addition to the RDT test in the field, a thick blood smear was collected, shipped and read at the Infectious Diseases Research Collaboration (IDRC) reference laboratory in Kampala during the PBO parasite surveys. At the reference laboratory, blood smears were stained with 2% Giemsa for 30 min. Each blood slide was read independently by two competent (WHO competency assessment level 1) laboratory scientists. The slide readers were blinded to each other’s results and were not aware of patients’ RDT results. Thick blood smears were evaluated for the presence of parasites (asexual forms) and gametocytes following standard WHO methodology [35]. Parasitaemia was determined by counting the number of parasites per 200 white blood cells (WBC), or 500 WBCs for low-density infections, on thick smears (assuming a standard of 8000 WBC per µl in accordance with WHO methods) [35]. Smears were considered negative if no parasites were seen in 200 oil-immersion fields (1000×) in a thick blood films. All blood smear results were obtained from the PBO study database. Blood smears were only retrieved for cross-checking and quality control purposes.

### Parasite DNA extraction

All DBSs for the *pfhrp2* and *pfhrp3* studies were shipped to the Australian Defence Force Malaria and Infectious Disease Institute (ADFMIDI) where molecular testing was conducted. From each DBS sample, three discs of DBS were punched into 1.5-mL microfuge tubes. DNA was extracted using QIAamp DNA Mini Kits and a QIAcube Robot (QIAGEN, Crawley, UK) according to the manufacturer’s instructions. Samples were eluted into a volume of 100 µl with AE buffer. A *P. falciparum*-positive control DBS spot was extracted and processed in each run alongside samples. Details of the QIAamp DNA Mini Kits and a QIAcube Robot extraction method has been described and published elsewhere [12, 17, 25, 36, 37].

### Confirmation of *Plasmodium falciparum* parasite DNA

Presence of different *Plasmodium* spp. was confirmed by amplification of the *18S ribosomal RNA* (18S rRNA) gene using multiplex PCR. Presence of *P. falciparum* infection was further confirmed by *P. falciparum*-specific PCR and amplification of the *msp1* and *msp2* single copy genes. Gel electrophoresis using 2% agarose was used to confirm the presence of bands. The detailed procedure for the controls, primers and the PCR conditions used have been described previously and widely published [12, 17, 25, 36, 37].

### Amplification of *pfhrp2* and *pfhrp3* parasite genes

All samples that were confirmed as *P. falciparum* positive and in which *msp1* and *msp2* were detected, the exon 1 and exon 2 of the *pfhrp2* and *pfhrp3* genes were amplified to investigate the presence or absence of *pfhrp2* and *pfhrp3* genes. PCR controls using laboratory lines DD2, 3BD5, HB3, and 3D7 with known *pfhrp2/3* status and human negative controls were included in each PCR run. PCR runs were only considered valid if all controls were amplified and resulted in bands of expected size on gel electrophoresis. The detailed procedures, primers used and PCR conditions have been well described and published elsewhere [12, 17, 25]. In all cases, samples were considered gene deleted if they had a positive *P. falciparum* DNA PCR and confirmed presence of *msp1* and *msp2* single copy genes but failed to amplify exon 1 or exon 2 of the *pfhrp2* or *pfhrp3* genes.

### Quality control

As part of quality control, all slides were read in a blinded manner by WHO-certified level 1 microscopists. In addition, a random sample of 20% of the slides were re-read by two level 1 WHO-certified microscopists and a third level 1 expert resolved any discrepant readings (differences between two microscopy readings including > 20% difference in parasite counts, or between RDTs and smears). All three slide readers were independent from an external laboratory. The research laboratory in Australia where molecular analysis of samples was done is a WHO Collaborating Centre for malaria, a member of the WHO *pfhrp2* and *pfhrp3* gene deletion detection laboratory network and participates in the WHO nucleic acid amplification tests (NAAT) external quality assurance programme.

### Ethical approval

The study was approved by the Makerere University School of Medicine Research and Ethics committee (#REC REF 2017-111), the Uganda National Council of Science and Technology (Ref No: HS271ES), and the Australian Department of Defence and Veterans’ Affairs Human Research Ethics Committee (DDVA HREC 096-18). In the primary surveys from where samples were collected, participants were enrolled after providing consent following a detailed explanation about the use of samples for future research studies.

### Statistical analysis

The aim was to estimate the proportion of *pfhrp2*/*pfhrp3* gene deletion in the parasite isolates to within 5 percentage points (absolute precision) of the true value with 95% confidence. The assumption that the prevalence of deletions in the *P. falciparum* isolates was unlikely to exceed 5% and a design effect of 1.5 were considered in order to estimate the minimum required sample.

As part of data management, demographics and predictor variables linked to the DBS samples were extracted from the primary PBO study database. All data were entered and managed in one central Excel database. Data quality checks were done to check for and correct any inconsistencies. Data analysis was done with STATA Ver 14, College Station, TX, USA: StataCorp LP). Descriptive analysis was done to describe the baseline characteristics with respect to the predictor variables. ArcGIS software version 10.8, Environmental Systems Research Institute (ESRI), CA, USA) was used to map the locations from where all blood samples were collected and where the *pfhrp2 and pfhrp3* gene deletions actually occurred. Bivariate analysis was performed to relate *pfhrp2/3* gene deletions and each of the independent variables. The exact binomial test was used to assess if the observed proportions of deletions were different from the 5%. As appropriate, the Chi square or Fisher’s exact test were used to compare proportions of deletions. Lastly, multivariate analysis was done with logistic regression to determine the factors associated with *pfhrp2/3* gene deletions. The 95% confidence interval was estimated for all estimates while *p *< 0.05 was considered significant.

## Results

### Characteristics of the study population

**Fig. 1:**
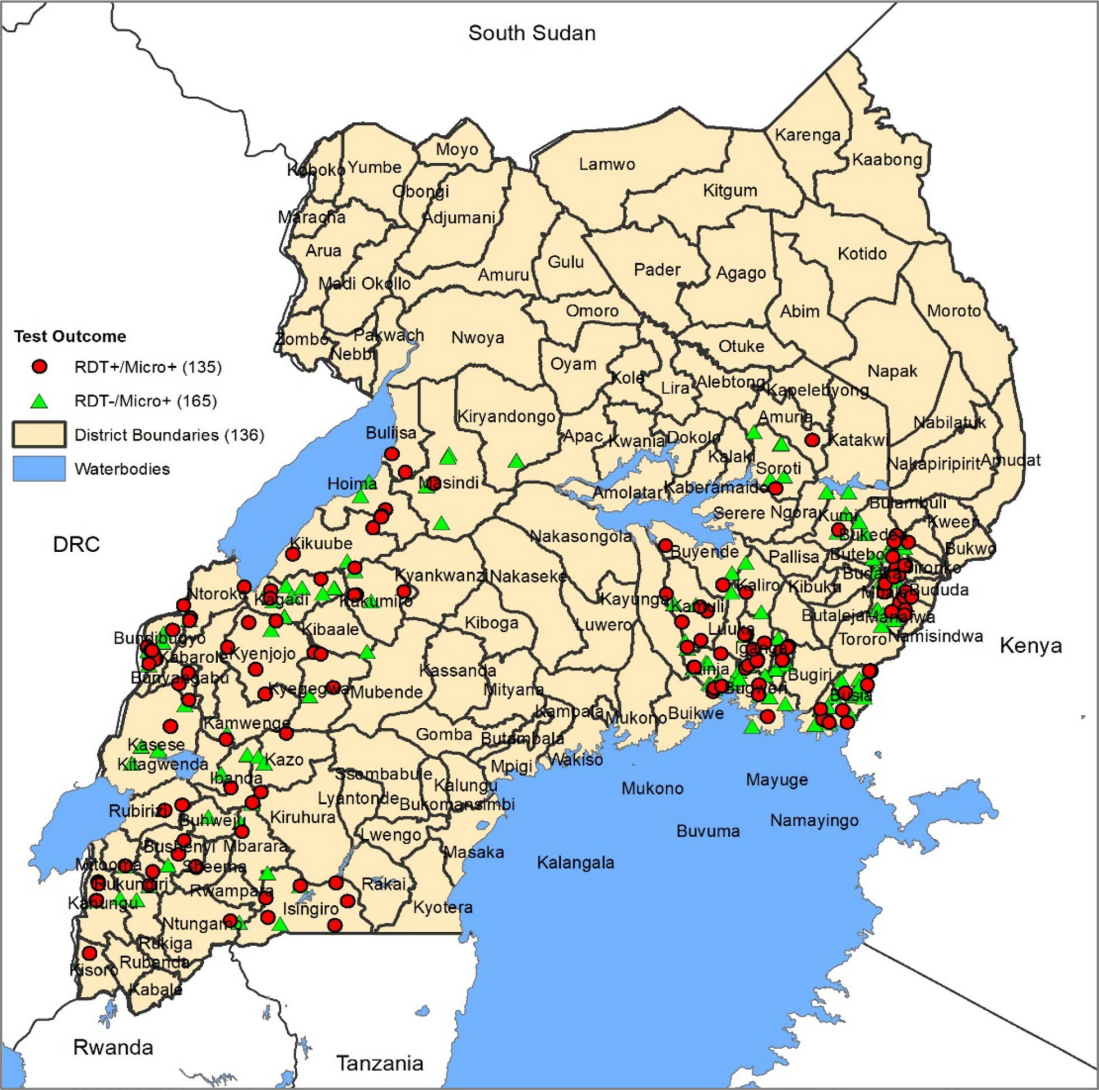
Geographical information system (GIS) mapping and geographical distribution of sites where *Plasmodium falciparum* isolates were collected. Showing the location of sites where study samples were collected across 48 districts in eastern and western regions of Uganda. RDT−/microscopy+ (indicated by red dots) are samples that were RDT negative but microscopy positive. RDT+/microscopy+ (indicated by green symbols) are samples that were positive on both RDTs and microscopy

**Fig. 2:**
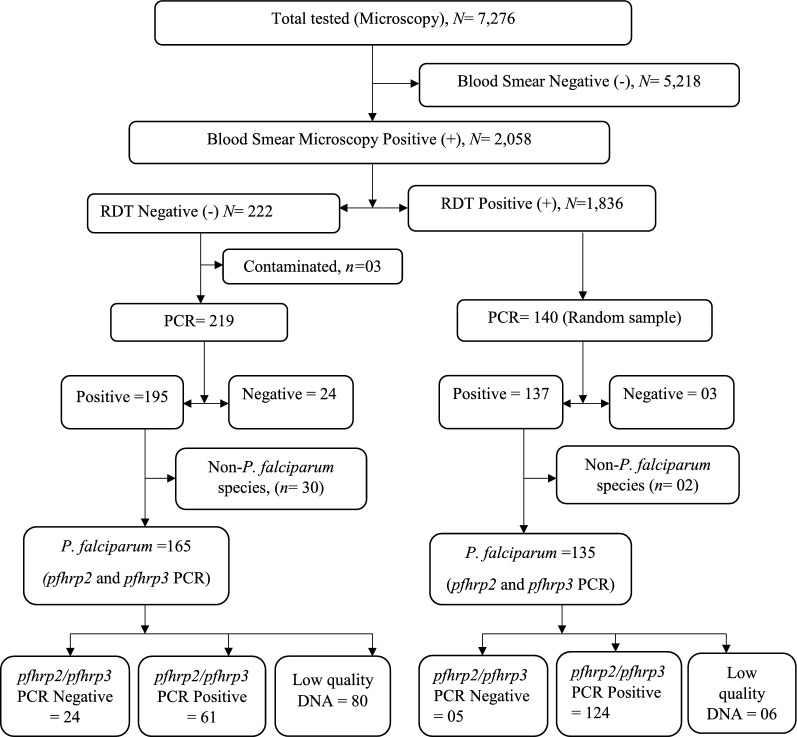
*pfhrp2* and *pfhrp3* study profile. RDT in this case means samples tested with HRP2 rapid diagnostic tests. PCR is the polymerase chain reaction for parasite detection and speciation. *pfhrp2 and pfhrp3* PCRs are the polymerase chain reactions for amplification of exon 1 and exon 2 of *P. falciparum* histidine-rich protein 2 and histidine-rich protein 3 genes. *pfhrp2/pfhrp3* PCR negative are samples in which *pfhrp2/3* genes were missing despite presence of parasite DNA and *msp1* and *msp2* single copy genes. Low quality DNA means samples that were DNA PCR positive but could not amplify two single copy genes (*msp1* and *msp2*) as indicator of quality of DNA

Out of 7276 participants enrolled and tested for malaria in the PBO surveys, 2058 (28.3%) had a positive blood smear. Of the 2058, 10.8% (222/2058) had a negative RDT despite a positive blood smear and were considered for the *pfhrp2/3* study. In addition, a random sample of 140 (i.e., 15%) of the RDT-positive/microscopy-positive samples (RDT+/microscopy+) were included for *pfhrp2/3* deletion study. Sixty-two samples (57 in the RDT-/micro+ and 5 in the RDT+/micro+) were excluded from gene deletion analysis due to contamination (n = 3), absence of parasite DNA (n = 27), and non-*P. falciparum* species (n = 32), leaving 300 samples for *pfhrp2/3* gene deletion analysis. The distribution of study samples (RDT−/microscopy+ and RDT+/microscopy+)  across the study sites is shown in Fig. 1 and the study profile in Fig. 2.

The majority of participants studied were male (52.3%) and were aged > 5 years (59.3%). Most participants were from the eastern region of Uganda 56.7% (50.9–62.4%). A majority of samples had parasite density ≥ 1000/µl Table 1.

**Table 1:** Baseline characteristics of samples (*n *= 300)

Variable	Frequency	Proportion (%)
Gender		
Male	156.90	52.30
Female	143.10	47.70
Age (years)		
< 5	122.10	40.70
≥ 5	177.90	59.30
Region		
Eastern	170.10	56.70
Western	129.90	43.30
Endemicity		
Low transmission	195.90	65.30
Moderate transmission	104.10	34.70
Parasite density (μL)		
< 1000	117.00	39.00
≥ 1000	183.00	61.00

< 5 means children under 5 years of age; ≥ 5 means children above 5 years of age. Low transmission means *P. falciparum* prevalence of ≤ 10% (≤ 10% P*f*PR), moderate transmission means (10–35% P*f*PR) based on WHO surveillance guidelines for malaria epidemiological stratification [38]; < 1000 and ≥ 1000 are parasite quantification counted per microlitre of blood

Overall, the *pfhrp2* and *pfhrp3* genes were deleted in 9.7% (29/300) of the *P. falciparum* isolates (95% CI 6.6–13.6%). The specific proportions of gene deletions were 3.3% (95% CI 1.6–6.0%) for *pfhrp2*−*/pfhrp3*+, 3.0% (95% CI 1.4–5.6%) *for pfhrp2*+*/pfhrp3*− and 3.3% (95% CI 1.6–6.0%) for *pfhrp2*−*/pfhrp3*−. The *pfhrp2* and *pfhrp3* genes were present and detected in 62.0% (186/300) of the *P. falciparum* isolates (95% CI 55.9–67.2%).

The proportion of gene deletions were significantly higher in RDT−/microscopy+ samples 14.5% (95% CI 9.5–20.9%) compared to the RDT+/microscopy+ samples 3.7% (95% CI 1.2–8.4%), *p *= 0.001. An important observation to note is that parasite densities were significantly lower in the RDT-/microscopy + compared to the RDT +/microscopy + samples (median: 520.0 (119.5–1086.5 vs 8400 (3628.5–29,600.0), p = 0.001.

The proportions of *P. falciparum* isolates with detectable *pfhrp2* and *pfhrp3* genes were significantly higher in the RDT+/microscopy+ , 91.9% (95% CI 85.9–95.9%) compared to the RDT−/microscopy+ samples, 37.0% (95% CI 29.6–44.8%) *p* = 0.001.

Overall, *pfhrp2* and *pfhrp3* gene deletions were higher in parasite isolates collected from eastern region, 14.7% (95% CI 9.7–20.9%) compared to western region of Uganda 3.1% (95% CI 0.8–7.7%), *p* = 0.001. The difference in this distribution was more marked when the parasites had both *pfhrp2* and *pfhrp3* deletions (*pfhrp2*−*/pfhrp3*−), 5.3% vs 0.8%, *p* = 0.047 for eastern and western regions, respectively (Table 2).

**Table 2:** Proportion of *pfhrp2* and *pfhrp3* gene deletion overall, by RDT−/microscopy+/PCR+, RDT+/microscopy+/PCR+ and by geographical location

Gene deletion	Overall proportions and stratified by RDT/microscopy results				
	Total (N = 300), Proportion n (%, 95% CI)	RDT-/micro+/PCR+ N = 165 n (%, 95% CI)	RDT+/micro+/PCR+ N = 135 n (%, 95% CI)	Prevalence ratio (RDT-/RDT+) n (95% CI)	*p* value
Any deletion	29 (9.7, 6.6–13.6)	24 (14.5, 9.5–20.9)	5 (3.7, 1.2–8.4)	3.9 (1.5–10.0)	0.002
*pfhrp2*−*/pfhrp3*+	10 (3.3, 1.6–6.0)	9 (5.5, 2.5–10.1)	1 (0.7, 0.0–4.1)	7.4 (1.0–57.4)	0.021
*pfhrp2 *+*/pfhrp3*−	9 (3.0, 1.4-5.6)	5 (3.0, 1.0–6.9)	4 (3.0, 0.8–7.4)	1.02 (0.3–3.7)	1.000
*pfhrp2*−*/pfhrp3*−	10 (3.3, 1.6–6.0)	10 (6.1, 2.9–10.9)	0 (0.0, 0.0–2.7)	N/A	0.004
*pfhrp2 *+*/pfhrp3*+	185 (61.7, 55.9–67.2)	61 (37.0, 29.6–44.8)	124 (91.9, 85.9–95.9)	0.4 (0.3–0.5)	0.001

Deletions by geographical location				
Gene deletion	Eastern (N = 170), n (%, 95% CI)	Western (N = 130), n (%, 95% CI)	Prevalence ratio (Eastern/Western) n (95% CI)	p-value
Any deletion	25 (14.7, 9.7–20.0)	4 (3.1, 0.8–7.7)	4.8 (1.7–13.4)	0.001
*pfhrp2*−*/pfhrp3*+	8 (4.7, 2.1–9.1)	2 (1.5, 0.2–5.4)	3.1 (0.7–14.2)	0.125
*pfhrp2*+*/pfhrp3*−	8 (4.7, 2.1–9.1)	1 (0.8, 0.0–4.2)	6.1 (0.8–48.3)	0.050
*pfhrp2*−*/pfhrp3*−	9 (5.3, 2.4–9.8)	1 (0.8, 0.0–4.2)	6.9 (0.9–53.6)	0.032
*pfhrp2*+*/pfhrp3*+	102 (60.0, 52.2–67.4)	83 (63.8, 55.0–72.1)	0.9 (0.8–1.1)	0.502

Parasite isolates were categorized as those in which the *pfhrp2* gene was deleted but *pfhrp3* gene present (*pfhrp2*−*/pfhrp3*+), the *pfhrp3* gene deleted but *pfhrp2* gene present (*pfhrp2*+*/pfhrp3*−), those in which both genes were deleted (*pfhrp2*−*/pfhrp3*−) and those where both *pfhrp2* and *pfhrp3* genes were present (*pfhrp2*+*/pfhrp3*+). Any deletion means total (overall) number of samples where deletion of any type was detected (summation of *pfhrp2*−*/pfhrp3*+*, pfhrp2*+*/pfhrp3*− and *pfhrp2*−*/pfhrp3*−)

**Fig. 3:**
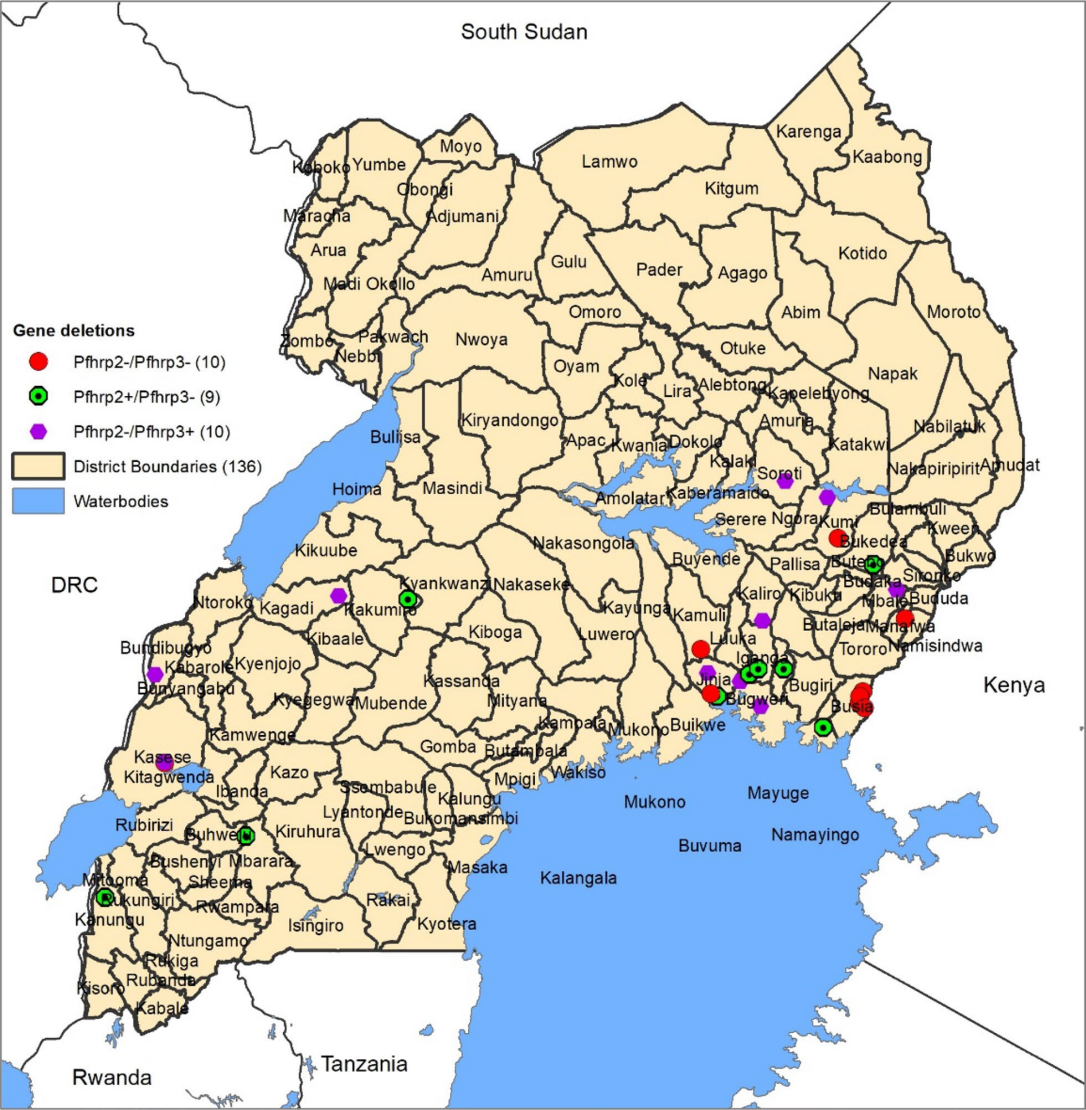
Mapping the exact locations of *pfhrp2* and *pfhrp3* gene-deleted *Plasmodium falciparum* isolates. Exact location of collection sites for 29 gene-deleted *P. falciparum* parasites by latitude and longitudes coordinates. *pfhrp2*−*/pfhrp3*+ (indicated by red dots), *pfhrp2*+*/pfhrp3*− (indicated by green circles) and *pfhrp2*-*/pfhrp3*− (indicated by the purple hexagons)

All 29 *P. falciparum* isolates with *pfhrp2/3* gene deletions were mapped based on latitude and longitude coordinates to determine their exact location in the study area Fig. 3. There was pronounced clustering of the *pfhrp2*−*/pfhrp3*− gene deleted isolates in mid-eastern Uganda and near the Uganda-Kenya border. The *pfhrp2*−*pfhrp3*+ isolates in western Uganda were mainly clustered along the Ugandan border with DRC (Fig. 3).

Nine different types of *pfhrp2* and *pfhrp3* gene deletion patterns were observed in the 29 *pfhrp2/3*-deleted isolates (Table 3). Out of 29 *P. falciparum* parasite isolates identified with gene deletions, 8 (27.6%) had complete deletion of the *pfhrp2/3* exon1 and exon2. The rest of the isolates had deletions of either exon1 or exon2 of *pfhrp2* and *pfhrp3* genes.

**Table 3:** Pattern of deletions in the *pfhrp2* and *pfhrp3* genes in the 29 deleted *Plasmodium falciparum* isolates (*n *= 29)

*P.f* DNA PCR	*msp1*	*msp2*	*pfhrp2* *Exon1*	*pfhrp2* *Exon*-*2*	*pfhrp3 Exon1*	*pfhrp3* *Exon*-*2*	Sample (%)
+	+	+	−	−	−	−	8 (27.6%)
+	+	+	−	−	−	+	5 (17.2%)
+	+	+	−	−	+	−	1 (3.4%)
+	+	+	−	−	+	+	2 (6.9%)
+	+	+	+	−	−	+	1 (3.4%)
+	+	+	+	−	+	+	3 (10.3%)
+	+	+	+	+	−	−	1 (3.4%)
+	+	+	+	+	−	+	2 (6.9%)
+	+	+	+	+	+	−	6 (20.7%)

PCR amplification and detection results for the 29 deleted samples. Positive and negative PCR results are represented by (+) and (−) respectively)

Additional data was obtained on predictor variables and statistical testing performed to determine if any were possibly associated with the *P. falciparum* gene deletions. The predictor variables included endemicity, geographical location, age, gender, parasite density of the samples per microlitre of blood (Table 4).

**Table 4:** Factors associated with *pfhrp2/3* deletions (overall)

Variable	Univariable		Multivariable	
	OR (95% CI)	p-value	aOR (95% CI)	p-value
Gender				
Male	1.00		1.00	
Female	1.20 (0.55–2.60)	0.646	1.24 (0.55–2.80)	0.598
Age (years)				
< 5	1.00		1.00	
≥ 5	1.34 (0.61–3.10)	0.477	1.52 (0.68–3.61)	0.321
Geographical location				
Eastern	5.43 (2.04–18.81)	0.002	6.25 (2.02–23.55)	0.003
Western	1.00		1.00	
Endemicity				
Low transmission	1.00		1.00	
Moderate transmission	1.88 (0.86–4.08)	0.109	0.78 (0.32–1.91)	0.579
Parasite density (μL)				
<1000	1.12 (0.50–2.41)	0.782	0.97 (0.42–2.16)	0.943
≥1000	1.00		1.00	

*pfhrp2*−*/pfhrp3*+ gene deletions				
Variable	Univariable		Multivariable	
	OR (95% CI)	p-value	aOR (95% CI)	p-value
Gender				
Male	1.00		1.00	
Female	1.10 (0.30–4.04)	0.881	0.89 (0.23–3.39)	0.862
Age (years)				
< 5	1.00		1.00	
≥ 5	1.62 (0.44–7.65)	0.489	1.64 (0.43–7.92)	0.493
Geographical location				
Eastern	3.16 (0.78–21.18)	0.15	6.84 (1.50–48.30)	0.022
Western	1.00		1.00	
Endemicity				
Low transmission	1.00		1.00	
Moderate transmission	0.46 (0.07–1.88)	0.333	0.19 (0.03–0.88)	0.049
Parasite density (μL)				
< 1000	0.66 (0.14–2.43)	0.555	0.61 (0.13–2.30)	0.488
≥ 1000	1.00		1.00	

Overall, deletions were more likely to occur in the eastern compared to western regions of Uganda, aOR 6.25 (95% CI 2.02–23.55), *p* = 0.003. When stratified the *pfhrp2*−*/pfhrp3*+gene deleted parasites were still more detectable in samples collected from eastern Uganda aOR 6.84 (1.50–48.30), *p* = 0.022.

In Uganda, malaria transmission is epidemiologically stratified according to the WHO surveillance guidelines into low (≤ 10% P*f*PR), moderate (10–35% P*f*PR) and high (≥ 35% P*f*PR) transmission based on population-based parasite surveys [38]. The *pfhrp2*−*/pfhrp3*+ gene deletions were less likely to occur in parasite isolates collected from moderate compared to low transmission settings aOR 0.19 (95% CI 0.03–0.88), *p *= 0.049. In this study, parasite density and gene deletions were not associated, aOR 0.97 (0.42–2.16), *p *= 0.943 as deletions occurred in both low and high parasite density samples.

## Discussion

This is the first large-scale survey reporting the presence of *pfhrp2* and *pfhrp3* gene deletions in *P. falciparum* parasite isolates in Uganda. The methods used in the study are adopted from the WHO-recommended protocol for investigation of *pfhrp2* and *pfhrp3* gene deletions [11]. Samples were confirmed for the presence of parasite DNA and gene deletion classifications were made following the WHO recommended procedure, i.e., quality assured DNA quality by amplifying single copy genes *msp1* and *msp2* before performing the *pfhrp2* and *pfhrp3* gene specific PCRs [11, 12]. These methods have been used and widely published in many studies [12, 16, 17, 25].

The study objectives were to determine the proportion of *pfhrp2/3* gene deletions in the parasite isolates, extent of spread and investigate the possible factors associated with these deletions. Overall, it was observed that the gene deletions were present in 9.7% (95% CI 6.6–13.6) of the *P. falciparum* parasite isolates in the exon1 and exon2 of *pfhrp2/3* genes. The gene deletions occurred in both surveyed regions but were disproportionately higher in eastern Uganda 14.7% (9.7–20.9), *p *= 0.001. The specific gene deletions were *pfhrp2(*−*)/pfhrp3(*+*)* 3.3% (CI 1.6–6.0), *pfhrp2(*+*)/pfhrp3(*−*)* 3.0% (CI 1.4–5.6) and *pfhrp2(*−*)/pfhrp3(*−*)* 3.3% (CI 1.6–6.0). A higher proportion of *pfhrp2* and *pfhrp3* gene deletions was observed in samples that were RDT negative but microscopy positive (RDT−/microscopy+), 14.5% (9.5–20.9%), *p *= 0.001. GIS mapping of parasite locations showed clustering of the gene deletions close to the Uganda-Kenya border in eastern Uganda and near the Uganda-DRC border in western Uganda (Fig. 3). Overall, a significant proportion of this *P. falciparum* parasite population contained the *pfhrp2* and *pfhrp3* genes 62.0% (55.9–67.2), *p *= 0.001.

The relatively low proportions of gene deletions observed in this study suggests that most parasite isolates were able to express HRP2 antigen (185/300) 62.0% and therefore HRP2-based RDTs will still be useful for malaria diagnosis in these areas. However, the fact that a proportion (24/300) of the *P. falciparum* isolates lacked the *pfhrp2/3* genes and evaded detection and subsequent treatment is of concern. In view of the fact that the HRP2-based RDTs are widely deployed in Uganda, the occurrence and confirmation of *pfhrp2/3* gene deletions in *P. falciparum* parasites may have implications for malaria case management and surveillance, particularly in areas where they have been mapped and located. It is important to conduct follow-up surveys to monitor their prevalence as recommended by the WHO [9, 11]. However, the proportion of gene deletions observed in Ugandan parasite isolates is lower than what was reported in Eritrea and Rwanda [17, 28]. It is however higher than the levels reported in Kenya, Tanzania, DRC, Ghana, and Mali [22–24, 26, 27, 30, 31, 39, 40]. The specific gene deletions of *pfhrp2(*−*)/pfhrp3*(+), *pfhrp2(*+*)/pfhrp3(*−*)* and *pfhrp2(*−*)/pfhrp3(*−*)* were generally lower compared to what has been reported in neighbouring countries. An important point to note however is that the comparison *pfhrp2* and *pfhrp3* findings across studies in Africa is challenging due to the wide variations in methods and computations of proportions using different denominators [12, 16]. Harmonization of methods for investigation of gene deletions based on WHO-recommended protocol will allow better comparison between studies [11, 16].

As expected, high proportions of gene deletions were observed in samples that were RDT negative but microscopy positive for malaria (RDT−/micro+) compared to those that were RDT and microscopy positive (RDT+/micro+). This indicates that gene deletions are one of the contributors to false negative RDT results in Uganda. However, the presence of non-*P. falciparum* species (*n *= 32) and low parasite densities as indicated by low quality DNA (*n *= 86) particularly in RDT-/microscopy + samples suggests that the two could have contributed to false negative RDTs. The contribution of gene deletion to false negative RDTs has been observed and reported elsewhere in previous studies [12, 17, 19, 22, 26–28, 30, 31]. The occurrence of fewer deletions in RDT +/micro + samples supports the assumption that the isolates still harbour the *pfhrp2* genes and are therefore able to express the HRP2 antigen. The WHO protocol recommends the RDT-/microscopy+ category as the most suitable samples for analysing gene deletions [10, 11]. However, in this study *P. falciparum* isolates with *pfhrp2/pfhrp3* gene deletions were also detected in the RDT+/microscopy+ category of samples, 3.7% (95% CI 1.2–8.4%). This observation supports the previous findings suggesting cross-reactivity between the HRP2 and HRP3 [11, 12, 16, 17, 25, 41]. The detection of *pfhrp2/pfhrp3* gene deletions in the RDT+/microscopy+ category of samples suggests the possibility of underestimation of the true proportions of deletion in studies that limit themselves to the RDT−/microscopy+ samples only.

Despite the occurrence of *P. falciparum* gene deletions in both surveyed regions, they were significantly higher in eastern Uganda, 14.7% (CI 9.7–20.9), *p *= 0.001. Using latitude and longitude coordinates, areas where all the *P. falciparum* gene-deleted isolates occurred were mapped and located. Although the gene-deleted parasites occurred across the two regions, they were more clustered close to the Uganda–Kenya border and in mid-eastern Uganda. The occurrence of gene deletions in the mid-eastern region had been reported previously in one district in 9 isolates (seven *pfhrp2* and two *pfhrp3* deletions) out of 416 PCR-confirmed samples and this study confirms this finding [32]. Some gene-deletion clustering was also observed near the Uganda-DRC boarder in western Uganda. Geographical clustering of *pfhrp2/pfhrp3*-deleted parasites could be explained by selection pressure as a result of selective treatment of RDT-positive *P. falciparum* parasites [30]. In view of these findings, the occurrence of deletions in both western and eastern regions of Uganda may have implications for future consideration of RDT deployment and establishment of surveillance systems. Epidemiologically, some parts of the mid-eastern region of Uganda have persistently reported a higher malaria burden, however clinical diagnosis and non-adherence to RDT results remains one of the highest in the country [4, 8]. Clinical diagnosis has poor specificity and may miss identification of true parasitaemic patients, which potentially allows survival and selective pressure of gene-deleted parasites [12, 17]. Follow-up studies should investigate the role of *pfhrp2/3* deletions in causing false negative RDTs, severe disease and sustaining malaria burden around this region. Koita et al. showed the potential of *pfhrp2/3* gene-deleted parasites to cause severe disease in Mali [22]. Regional and geographical variations in proportions of gene deletions observed in this study are consistent with what has been reported elsewhere in DRC, India and Eritrea [13, 16, 17, 30]. In Eritrea, *pfhrp2/3* deletion varied between hospitals in different locations of the country [17]. In DRC and India, the proportions of gene deletions varied across provinces and states [13, 30]. Geographical clustering of *pfhrp2* and *pfhrp3* gene-deleted parasites was reported in malaria-endemic regions of eastern DRC and western Kenya suggesting a possibility of cross-border transmission [30, 31]. The gene deletion mapping data obtained in this study could inform better targeting of *pfhrp2/3* follow-up surveys to monitor the levels of deletions. WHO recommends the establishment of surveillance systems that particularly focus on catchment areas where deletions have been reported and where false RDTs results are being reported [11, 16].

The study explored the possible factors associated with gene deletion in this study. As reported elsewhere, geographical location was an important factor for gene deletion [30]. The deletions were more likely to be detected in the eastern compared to the western region, aOR 6.25 (95% CI 2.02–23.55), *p *= 0.003. This observation was seen in similar studies in the DRC, India and Eritrea [13, 17, 30]. The association between geographical location and gene deletion may be explained by the evolutionary mechanism of migration or spontaneous occurrence of genetic events in parasites in a specific locality [17, 19, 25, 42, 43]. Although malaria endemicity and overall gene deletion were not associated, aOR 0.78 (95% CI 0.32–1.91) *p *= 0.579, the *pfhrp2*-*/pfhrp3*+ gene deletions were less likely to be found in moderate- compared to low-transmission areas aOR 0.19 (95% CI 0.03–0.88), *p *= 0.049. A similar observation was reported by Koita et al. in Mali and Berhari et al. in Eritrea [17, 22]. This observation suggests that *pfhrp2*/*pfhrp3* deleted parasites may be easier to detect in areas of low transmission intensity where polyclonal infections or co-infection with wild-type parasites that could trigger a positive RDT and mask the presence of a *pfhrp2* deleted parasite are less likely to occur [12]. The increased risk of gene deletion in parasite isolates in low transmission settings has been explained by reduced multiclonal infections so that parasites with gene deletions are likely single clone infections and can be detected readily by the PCR method [12, 17]. The implication here is that in high transmission settings such as most parts of Uganda where multiclonal infections and co-infections are common will require robust and novel diagnostic tools to investigate gene deletions.

However, this study was not free from limitations. The study was not able to explore and investigate clinical correlates between the *pfhrp2/pfhrp3* infected and the naturally occurring wild type *P. falciparum* parasites due to limited clinical data available. It is important that follow-up studies explore to understand the virulence and pathogenicity of *pfhrp2/3* deleted parasites particularly in causing severe disease and if these parasites show different drug susceptibility patterns to the current anti-malarial medicines. Also, other known possible causes of false negative RDTs [12] were not explored in this study that could be considered in follow-up surveys.

Furthermore, the study was limited by the fact that the *P. falciparum* isolates analysed were obtained from only two regions of Uganda, which leaves the status of *pfhrp2/3* gene deletions in other regions unknown. It is recommended that future surveillance programmes should consider a more representative sample covering all regions of Uganda. It is equally important to note that the initial surveys where the *P. falciparum* isolates were obtained were not specifically designed for the *pfhrp2/3* gene surveillance, which could have had impact on the characterization and selection of samples.

It is important to recognize the difficulties associated with detection of deletions in multiclonal and co-infections with wild-type parasites that could trigger a positive RDT and mask the presence of a *pfhrp2*/3-deleted parasite strain. In this case the HRP2-based RDTs will be positive based on the wild-type parasite that is able to express HRP2 antigen, while the masked *pfhrp2/3* gene-deleted parasite remains undetected causing underestimation of deletions. However, this has been a challenge in many published studies due to limitation with the currently available molecular tools [12, 30].

While all the RDT−/microscopy+ samples were included in the analysis, only a random sub-set of the RDT+/microscopy+ was included on assumption that all the other (RDT+/microscopy+) samples contained parasites that expressed the HRP2 antigen. However, it is difficult to ascertain whether the RDT positivity was due to HRP2 or HRP3 expression since the two protein antigens share common epitopes due to high degree of similarity in amino acid sequence. This could have been a potential underestimation of *pfhrp3* deletion [12, 13].

Despite PCR confirmation, some samples in the RDT−/microscopy+ sub-set had low-quality DNA and could not amplify the two single copy genes probably due to low parasite densities. As recommended, such samples were not included for *pfhrp2/3* gene amplification, which could have led to possible underestimation of deletions [11, 12, 16, 29].

## Conclusions

This study provides the first evidence on a large scale of the presence of *pfhrp2* and *pfhrp3* gene deletions in *P. falciparum* isolates in Uganda. Deletions occurred in both the eastern and western regions of Uganda but were more marked in the east. Proportions of gene deletions were higher in (RDT−/microscopy−) samples compared to (RDT+/microscopy+). In view of these findings the roll-out of RDTs for malaria diagnosis will need to take into consideration the *pfhrp2/3* gene deletions in these regions. Periodic *pfhrp2/3* surveys will be important to inform future decisions for deployment of alternative RDTs in Uganda.

## Data Availability

Data for this *pfhrp2* and *pfhrp3* study is available and kept at the study central excel database and in safe custody.

## Abbreviations

HRP2: Histidine rich protein 2
*pfhrp2*: The *Plasmodium falciparum* histidine rich protein 2 gene
RDTs: Rapid diagnostic tests
ACT: Artemisinin-based combination therapy
MSP1: Merozoite surface antigen 1
MSP2: Merozoite surface antigen 2
PCR: Polymerase chain reaction
WHO: World Health Organization
ITNs: Insecticide treated mosquito nets
LLINs: Long-lasting insecticide-treated nets
IRS: Indoor residual spraying
GIS: Geographical Information System
DNA: Deoxyribonucleic acid
rRNA: Ribosomal ribonucleic acid
DBS: Dried blood spots
WBC: While blood cells
SNPs: Single nucleotide polymorphisms
NAAT: Nucleic acid amplification tests

## References

[1] WHO. World Malaria Report. Geneva, World Health Organization, 2019. https://www.who.int/publications-detail/world-malaria-report-2019.

[2] WHO. World Malaria Report. Geneva, World Health Organization, 2018. https://www.who.int/malaria/publications/world-malaria-report-2018/en/.

[3] NMCP. Uganda Malaria Indicator Survey (MIS) https://dhsprogram.com/pubs/pdf/ATR21/ATR21.pdf, 2019.

[4] MIS. Uganda Malaria Indicator Survey, https://dhsprogram.com/pubs/pdf/MIS21/MIS21.pdf. 2014.

[5] Talisuna AO, Mundia CW, Otieno V, Mitto B, Amratia P, Snow RW, et al. An epidemiological profile of malaria and its control in Uganda. https://files.givewell.org/files/DWDA%202009/Interventions/Nets/Resistance/NMCP_Uganda.pdf. 2013.

[6] WHO. Guidelines for treatment of malaria. 3rd Edn. 2015. https://www.who.int/docs/default-source/documents/publications/gmp/guidelines-for-the-treatment-of-malaria-eng.pdf?sfvrsn=a0138b77_2.

[7] NMCP. Uganda malaria control policy https://www.severemalaria.org/sites/mmv-smo/files/content/attachments/2017-02-28/Uganda%20NATIONAL%20MALARIA%20CONTROL%20POLICY%20-%202011.pdf. 2011.

[8] DHIS2 M. Routine district health information management system, Uganda. 2020.

[9] WHO. False-negative RDT results and implications of new *P. falciparum* histidine-rich protein 2/3 gene deletions. Geneva; World Health Organization, https://apps.who.int/iris/bitstream/handle/10665/258972/WHO-HTM-GMP-2017.18-eng.pdf. 2016.

[10] WHO. Malaria policy advisory committee meeting, 14–16 September 2016, Background document for Session 7; *P. falciparum hrp2*/3, gene deletions, conclusions and recommendations of a Technical Consultation Geneva, Switzerland, http://www.who.int/malaria/mpac/mpac-sept2016-hrp2-consultation-short-report-session7.pdf. Accessed 18 July 2016; 2016.

[11] WHO. Protocol for estimating the prevalence of pfhrp2/pfhrp3gene deletions among symptomatic falciparum patients with false-negative RDT results. 2018.

[12] Cheng Q, Gatton ML, Barnwell J, Chiodini P, McCarthy J, Bell D (2014). *Plasmodium falciparum* parasites lacking histidine-rich protein 2 and 3: a review and recommendations for accurate reporting. Malar J..

[13] Bharti PK, Chandel HS, Ahmad A, Krishna S, Udhayakumar V, Singh N (2016). Prevalence of pfhrp2 and/or pfhrp3 gene deletion in *Plasmodium falciparum* population in eight highly endemic states in India. PLoS ONE.

[14] WHO. Methods manualfor product testingof malaria rapid diagnostic test, Geneva; World Health Organization, 2018. https://www.who.int/malaria/publications/rdt-method-manual-product-testing.pdf?ua=1.

[15] Cunningham J, Jones S, Gatton ML, Barnwell JW, Cheng Q, Chiodini PL (2019). A review of the WHO malaria rapid diagnostic test product testing programme (2008–2018): performance, procurement and policy. Malar J..

[16] Agaba BB, Yeka A, Nsobya S, Arinaitwe E, Nankabirwa J, Opigo J (2019). Systematic review of the status of pfhrp2 and pfhrp3 gene deletion, approaches and methods used for its estimation and reporting in *Plasmodium falciparum* populations in Africa: review of published studies 2010–2019. Malar J..

[17] Berhane A, Anderson K, Mihreteab S, Gresty K, Rogier E, Mohamed S (2018). Major threat to malaria control programs by *Plasmodium falciparum* lacking histidine-rich protein 2, Eritrea. Emerg Infect Dis..

[18] Deme AB, Park DJ, Bei AK, Sarr O, Badiane AS, Gueye PH (2014). Analysis of pfhrp2 genetic diversity in Senegal and implications for use of rapid diagnostic tests. Malar J..

[19] Baker J, McCarthy J, Gatton M, Kyle DE, Belizario V, Luchavez J (2005). Genetic diversity of P*lasmodium falciparum* histidine-rich protein 2 (PfHRP2) and its effect on the performance of PfHRP2-based rapid diagnostic tests. J Infect Dis.

[20] Wurtz N, Fall B, Bui K, Pascual A, Fall M, Camara C (2013). Pfhrp2 and pfhrp3 polymorphisms in *Plasmodium falciparum* isolates from Dakar, Senegal: impact on rapid malaria diagnostic tests. Malar J..

[21] Baker J, Ho MF, Pelecanos A, Gatton M, Chen N, Abdullah S (2010). Global sequence variation in the histidine-rich proteins 2 and 3 of *Plasmodium falciparum*: implications for the performance of malaria rapid diagnostic tests. Malar J..

[22] Koita OA, Doumbo OK, Ouattara A, Tall LK, Konare A, Diakite M (2012). False-negative rapid diagnostic tests for malaria and deletion of the histidine-rich repeat region of the hrp2 gene. Am J Trop Med Hyg.

[23] Kumar N, Pande V, Bhatt RM, Shah NK, Mishra N, Srivastava B (2013). Genetic deletion of HRP2 and HRP3 in Indian *Plasmodium falciparum* population and false negative malaria rapid diagnostic test. Acta Trop.

[24] Menegon M, L’Episcopia M, Nurahmed AM, Talha AA, Nour BYM, Severini C (2017). Identification of *Plasmodium falciparum* isolates lacking histidine-rich protein 2 and 3 in Eritrea. Infect Genet Evol..

[25] Gamboa D, Ho MF, Bendezu J, Torres K, Chiodini PL, Barnwell JW (2010). A large proportion of *P falciparum* isolates in the Amazon region of Peru lack pfhrp2 and pfhrp3: implications for malaria rapid diagnostic tests. PLoS ONE.

[26] Gupta H, Matambisso G, Galatas B, Cistero P, Nhamussua L, Simone W (2017). Molecular surveillance of pfhrp2 and pfhrp3 deletions in *Plasmodium falciparum* isolates from Mozambique. Malar J..

[27] Funwei R, Nderu D, Nguetse CN, Thomas BN, Falade CO, Velavan TP (2019). Molecular surveillance of pfhrp2 and pfhrp3 genes deletion in *Plasmodium falciparum* isolates and the implications for rapid diagnostic tests in Nigeria. Acta Trop.

[28] Kozycki CT, Umulisa N, Rulisa S, Mwikarago EI, Musabyimana JP, Habimana JP (2017). False-negative malaria rapid diagnostic tests in Rwanda: impact of *Plasmodium falciparum* isolates lacking hrp2 and declining malaria transmission. Malar J..

[29] WHO. Surveillance template protocol for pfhrp2/pfhrp3gene deletions. Geneva, World Health Organization, 2020. https://apps.who.int/iris/bitstream/handle/10665/331196/9789240002036-eng.pdf?ua=1.

[30] Parr JB, Verity R, Doctor SM, Janko M, Carey-Ewend K, Turman BJ (2017). Pfhrp2-deleted *Plasmodium falciparum* parasites in the Democratic Republic of the Congo: a national cross-sectional survey. J Infect Dis.

[31] Beshir KB, Sepulveda N, Bharmal J, Robinson A, Mwanguzi J, Busula AO (2017). *Plasmodium falciparum* parasites with histidine-rich protein 2 (pfhrp2) and pfhrp3 gene deletions in two endemic regions of Kenya. Sci Rep..

[32] Thomson KBB, Cunningham J, Baiden F, Bharmal J, Katia J, Catherine MS (2019). pfhrp2 and pfhrp3 gene deletions that affect malaria rapid diagnostic tests for *Plasmodium falciparum:* analysis of archived blood samples from 3 African countries. J Infect Dis.

[33] Rugnao S, Gonahasa S, Maiteki CM, Opigo J, Yeka A, Katureebe A (2019). LLIN Evaluation in Uganda Project (LLINEUP): factors associated with childhood parasitaemia and anaemia 3 years after a national long-lasting insecticidal net distribution campaign: a cross-sectional survey. Malar J..

[34] Gonahasa S, Maiteki CM, Rugnao S, Dorsey G, Opigo J, Yeka A (2018). LLIN Evaluation in Uganda Project (LLINEUP): factors associated with ownership and use of long-lasting insecticidal nets in Uganda: a cross-sectional survey of 48 districts. Malar J..

[35] WHO. Malaria microscopy quality assurance manual. Geneva, World Health Organization, 2016. https://www.who.int/docs/default-source/documents/publications/gmp/malaria-microscopy-quality-assurance-manual.pdf?sfvrsn=dfe54d47_2.

[36] Rubio JM, Benito A, Roche J, Berzosa PJ, Garcia ML, Mico M (1999). Semi-nested, multiplex polymerase chain reaction for detection of human malaria parasites and evidence of *Plasmodium vivax* infection in Equatorial Guinea. Am J Trop Med Hyg.

[37] Padley D, Moody AH, Chiodini PL, Saldanha J (2003). Use of a rapid, single-round, multiplex PCR to detect malarial parasites and identify the species present. Ann Trop Med Parasitol.

[38] WHO. Malaria surveillance, monitoring & evaluation: a reference manual. Geneva, World Health Organization, 2018. https://apps.who.int/iris/bitstream/handle/10665/272284/9789241565578-eng.pdf?ua=1, 2018.

[39] Nderu D, Kimani F, Thiong’o K, Akinyi M, Karanja E, Meyer CG (2018). PfHRP2-PfHRP3 diversity among Kenyan isolates and comparative evaluation of PfHRP2/pLDH malaria RDT with microscopy and nested PCR methodologies. Parasitol Int.

[40] Amoah LE, Abankwa J, Oppong A (2016). *Plasmodium falciparum* histidine rich protein-2 diversity and the implications for PfHRP 2: based malaria rapid diagnostic tests in Ghana. Malar J..

[41] CDC report. Molecular surveillance for HRP2 and HRP3 gene expression in *Plasmodium falciparum* parasites from south and central America. Centers for Disease Control and Prevention, *P. falciparu*m pfhrp2 and pfhrp3 surveillance project, July 2012.

[42] Dorado EJ, Okoth SA, Montenegro LM, Diaz G, Barnwell JW, Udhayakumar V (2016). Genetic characterisation of *Plasmodium falciparum* Isolates with deletion of the pfhrp2 and/or pfhrp3 genes in Colombia: the Amazon Region, a challenge for malaria diagnosis and control. PLoS ONE.

[43] Akinyi S, Hayden T, Gamboa D, Torres K, Bendezu J, Abdallah JF (2013). Multiple genetic origins of histidine-rich protein 2 gene deletion in *Plasmodium falciparum* parasites from Peru. Sci Rep..

